# Population genomic structure of the gelatinous zooplankton species *Mnemiopsis leidyi* in its nonindigenous range in the North Sea

**DOI:** 10.1002/ece3.5468

**Published:** 2019-09-30

**Authors:** Christophe Verwimp, Lies Vansteenbrugge, Sofie Derycke, Thomas Kerkhove, Hilde Muylle, Olivier Honnay, Tom Ruttink, Isabel Roldán‐Ruiz, Kris Hostens

**Affiliations:** ^1^ Animal Sciences Unit Flanders Research Institute for Agriculture, Fisheries and Food (ILVO) Oostende Belgium; ^2^ Plant Sciences Unit Flanders Research Institute for Agriculture, Fisheries and Food (ILVO) Melle Belgium; ^3^ Department of Biology, Plant Conservation and Population Biology University of Leuven (KUL) Heverlee Belgium; ^4^ Marine Biology Research Group Ghent University Gent Belgium; ^5^ Department of Plant Biotechnology and Bioinformatics Ghent University Zwijnaarde Belgium

**Keywords:** genotyping‐by‐sequencing, invasion biology, *Mnemiopsis leidyi*, population differentiation, population genomics, Single nucleotide polymorphisms

## Abstract

Nonindigenous species pose a major threat for coastal and estuarine ecosystems. Risk management requires genetic information to establish appropriate management units and infer introduction and dispersal routes. We investigated one of the most successful marine invaders, the ctenophore *Mnemiopsis leidyi*, and used genotyping‐by‐sequencing (GBS) to explore the spatial population structure in its nonindigenous range in the North Sea. We analyzed 140 specimens collected in different environments, including coastal and estuarine areas, and ports along the coast. Single nucleotide polymorphisms (SNPs) were called in approximately 40 k GBS loci. Population structure based on the neutral SNP panel was significant (*F*_ST_ .02; *p* < .01), and a distinct genetic cluster was identified in a port along the Belgian coast (Ostend port; pairwise *F*_ST_ .02–.04; *p* < .01). Remarkably, no population structure was detected between geographically distant regions in the North Sea (the Southern part of the North Sea vs. the Kattegat/Skagerrak region), which indicates substantial gene flow at this geographical scale and recent population expansion of nonindigenous *M. leidyi*. Additionally, seven specimens collected at one location in the indigenous range (Chesapeake Bay, USA) were highly differentiated from the North Sea populations (pairwise *F*_ST_ .36–.39; *p* < .01). This study demonstrates the utility of GBS to investigate fine‐scale population structure of gelatinous zooplankton species and shows high population connectivity among nonindigenous populations of this recently introduced species in the North Sea.

**OPEN RESEARCH BADGES:**

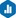

This article has earned an Open Data Badge for making publicly available the digitally‐shareable data necessary to reproduce the reported results. The data is available at: The DNA sequences generated for this study are deposited in the NCBI sequence read archive under SRA accession numbers SRR6950721–SRR6950884, and will be made publically available upon publication of this manuscript.

## Graphical abstract

The population genomic structure of the gelatinous zooplankton species *Mnemiopsis leidyi* in its nonindigenous range in the North Sea was investigated. Two genetically distinct clusters were identified.

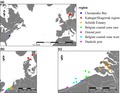

## 1 INTRODUCTION

Invasive species are widely recognized for their negative effects on biodiversity and ecosystem functioning (Carlton & Geller, 1993; Simberloff et al., 2013). During the last decades, globalization of maritime traffic has increased invasion rates of marine organisms by facilitating dispersal over large geographical distances (Hulme, 2009; Ricciardi & MacIsaac, 2000; Ruiz, Fofonoff, Carlton, Wonham, & Hines, 2000). Although only a fraction of the introduced species is able to thrive in a new environment, establishment of permanent populations can have dramatic effects on the local community (Katsanevakis et al., 2014; Molnar, Gamboa, Revenga, & Spalding, 2008; Ojaveer et al., 2015). Therefore, management of nonindigenous species should be a priority for marine conservation. Effective control measures and impact prediction rely on an accurate understanding of dispersal and population connectivity, which can be investigated with genetic approaches (Allendorf, Hohenlohe, & Luikart, 2010; Chown et al., 2015; Sherman et al., 2016; Viard, David, & Darling, 2016). Moreover, genetic reconstruction of invasion histories provides the opportunity to study the eco‐evolutionary mechanisms underlying long‐distance dispersal, range expansion, and local adaptation (Cristescu, 2015; Sax et al., 2007).

Marine invasions often affect valuable coastal and estuarine ecosystems (Grosholz, 2002). Marine species typically show limited population differentiation, and establishing appropriate management units is challenging. Estimating population connectivity is complicated by many factors specific to the marine environment (Palumbi, 2003). Obvious physical barriers to migration are seemingly absent, and habitat connectivity and high mobility promote long‐distance dispersal (Allendorf et al., 2010; Cowen & Sponaugle, 2009). Distribution ranges and population densities are shaped by seascape features such as ocean currents and physicochemical fluctuations and temperature (Hohenlohe, 2004; Johansson et al., 2015; O'Connor et al., 2007; Selkoe et al., 2016), and demographic processes such as local recruitment (Jones, Planes, & Thorrold, 2005). Moreover, the dispersal capabilities of pelagic organisms can be affected by other factors such as antropogenic pollution (Puritz & Toonen, 2011). Marine invertebrate species typically have a high reproductive output, and large population sizes prevent the accumulation of neutral divergence by genetic drift (Deagle, Faux, Kawaguchi, Meyer, & Jarman, 2015). Traditional genetic methods may lack the resolution to identify genetically differentiated populations. In contrast, genotyping‐by‐sequencing (GBS) allows identification of SNPs at thousands of loci to investigate fine‐scale population structure and accurate population assignment in the context of weak genetic structure (Andrews, Good, Miller, Luikart, & Hohenlohe, 2016; Davey et al., 2011; Narum, Buerkle, Davey, Miller, & Hohenlohe, 2013).

One of the most successful marine invaders is the ctenophore *Mnemiopsis leidyi*. This species is native to the Atlantic coasts of North and South America but is nowadays widespread across European seas. Introduction of *M. leidyi* in coastal ecosystems can induce community trophic cascades (Tiselius & Møller, 2017), and outbreaks have coincided with alarming changes in the pelagic food web (Oguz, Fach, & Salihoglu, 2008; Shiganova & Bulgakova, 2000). The invasive success of *M. leidyi* is attributed to its broad tolerance for environmental variability (Fuentes et al., 2010; Purcell, Shiganova, Decker, & Houde, 2001), flexible planktivorous diet (Costello, Bayha, Mianzan, Shiganova, & Purcell, 2012; Costello, Sullivan, Gifford, Van Keuren, & Sullivan, 2006; Rapoza, Novak, & Costello, 2005), and high fertility (Costello et al., 2012; Jaspers, Møller, & Kiørboe, 2015). Individuals are simultaneous hermaphrodite and capable of self‐fertilization. Maturity can be reached in a few weeks, after which thousands of eggs per day can be released (Jaspers, Costello, & Colin, 2014). *Mnemiopsis leidyi* is pelagic through its entire life cycle (Rapoza et al., 2005), which possibly promotes long‐distance dispersal via ocean currents. Molecular studies have identified distinct genetic clusters in Southern Europe (Black, Caspian and Mediterranean Sea) and Northwestern Europe (Baltic and North Sea; Bayha et al., 2015; Ghabooli et al., 2011; Reusch, Bolte, Sparwel, Moss, & Javidpour, 2010). These originate from distinct introductions that can be linked to the climatic conditions in the indigenous range. The Southern European cluster originates from a limited number of founders from the Gulf of Mexico, and subsequent outbreaks along the coast suggest a stepping‐stone scenario of colonization (Bolte et al., 2013; Fuentes et al., 2010; Ghabooli et al., 2013). The Northern European cluster originates from the Atlantic coast of North America. Possibly large numbers of ctenophores have been introduced by recurrent ballast water discharges, since no founder effects were detected (Bayha et al., 2015; Ghabooli et al., 2011; Reusch et al., 2010). However, these studies were based on a limited number of genetic markers and provided limited geographical resolution for risk assessment of contemporary outbreaks in Northwestern Europe. In the indigenous range, coastal embayments and estuaries support overwintering populations that populate adjacent coastal areas (Costello et al., 2006). Similar source–sink dynamics are expected in the nonindigenous range in Northwestern Europe (Collingridge, Molen, & Pitois, 2014; Schaber et al., 2011; Vansteenbrugge, Ampe, Troch, Vincx, & Hostens, 2015).

In the current study, we used high‐density SNP markers to investigate the spatial population structure of *M. leidyi* on three geographical scales. First, we estimated fine‐scale population differentiation and connectivity within the Southern part of the North Sea. Sampling locations covered three potential source populations (the ports of Dunkirk and Ostend, and the estuary of the Scheldt River) and potential sink populations along the Belgian coastal zone. We investigated the presence of distinct source populations and estimated their contribution to the coastal population. Based on source–sink dynamics, we expected to find higher genetic diversity in the three putative source populations compared with the population(s) in the North Sea. Second, we compared geographically distant regions; the Southern part of the North Sea and the Kattegat/Skagerrak region (DK) in the north. Third, sampling covered one location in the indigenous species range (Chesapeake Bay, USA). We expected low levels of population structure among the regions in the Southern part of the North Sea, due to local, annual migration, and increasing genetic differences between individuals from the Southern part of the North Sea, the Baltic Sea, and Chesapeake Bay due to isolation by distance. Our main aims were to develop a GBS procedure for *M. leidyi* with a focus on marker density and data completeness, and to describe spatial variation in SNP frequencies after the introduction and secondary spread in the North Sea. Specific goals were to (a) identify outlier SNPs putatively under natural selection; (b) determine the population structure based on all SNPs and neutral SNPs separately; (c) quantify genomewide diversity and structure among environmentally distinct regions in the Southern part of the North Sea and the geographically distant regions of Kattegat/Skagerrak (nonindigenous) and Chesapeake Bay (indigenous); and (d) identify recent migrants between regions by population assignment.

## 2 MATERIAL AND METHODS

### 2.1 Sample collection

**Figure 1:**
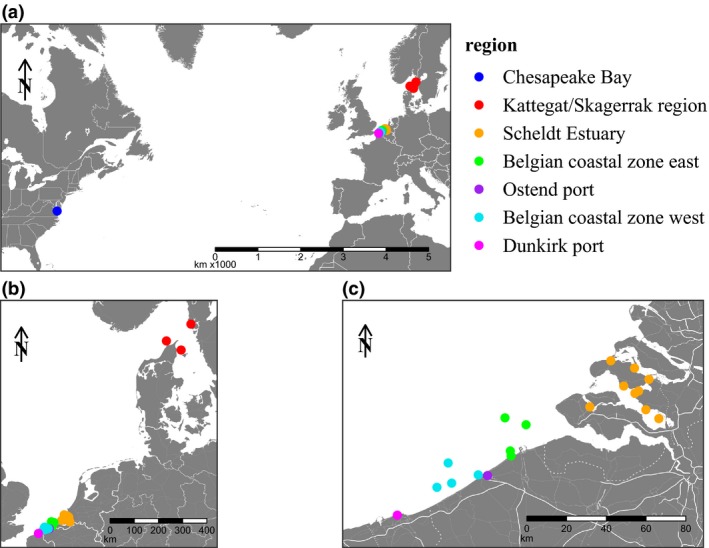
Geographical distribution of sampling locations covers both geographically distant and environmentally distinct regions. (a) Transatlantic range, including one sampling location in *M. leidyi's* indigenous range. (b) North Sea range, including three sampling location in the Kattegat/Skagerrak region. (c) Southern part of the North Sea, including the Belgian coast and potential source populations in the ports of Dunkirk and Ostend, and the Scheldt Estuary (Table 1)

*Mnemiopsis leidyi* specimens were collected from 23 sampling locations (Figure 1). Several stations along the Belgian coast, in the port of Ostend and in the Scheldt estuary were sampled using plankton net trawling (both CalCOFI net and hyperbenthic sledge towed at three knots, mesh size 1,000 µm), scuba diving, or dip‐net sampling from June until November 2014 (Table 1). Specimens of the port of Ostend were collected in two subsequent years (2014 and 2015; Table 1). Additionally, specimens were collected by plankton net trawling: in the port of Dunkirk (FR) in 2012 and 2013, the Kattegat/Skagerrak region (DK) in 2014, and Chesapeake Bay (USA) in 2013. Specimens were preserved individually in 99% ethanol.

**Table 1:** Overview and details of the sampling locations, dates, and geographic regions

Region	Individual samples (*n*)	Location	Latitude	Longitude	Sampling date and individual samples (*n*)												
					2012			2013		2014						2015	
					8	9	10	8	9	6	7	8	9	10	11	4	5
Chesapeake Bay (*)	6	Chesapeake Bay	37.5704	−76.1718				7									
Kattegat/Skagerrak	16	Gullmar Fjord	58.2490	11.4000											4		
		Kattegat	57.4256	10.8143									4				
		Skagerrak	57.7163	9.9361									8				
Scheldt estuary	27	OS5	51.5220	4.0826	2												
		OS6	51.4821	4.1762	3												
		OS9	51.6057	4.0310	1												
		Eastern Scheldt 1	51.6277	3.9240									2				
		Grevelingen 1	51.7402	3.8308									4		5		
		Grevelingen 2	51.7078	4.0013								1			1		
		Mastgat 1	51.6577	4.1052									2				
		Mastgat 2	51.5953	4.0041											2		
		Veerse Meer 1	51.5347	3.6801						4							
Belgian coastal zone east	44	Diep1	51.4857	3.0700								2	5				
		Knok1	51.4548	3.2218								4	2				
		Zee1	51.3375	3.1086								4	6	16			
		Blankenberge	51.3150	3.1140									5				
Ostend port	12	Ostend	51.2269	2.9449						2	2					4	4
Belgian coastal zone west	28	Oost1	51.2299	2.8777									2	6			
		Kwint	51.2833	2.6617									9				
		West1	51.1929	2.6869									5				
		West2	51.1740	2.5810									6				
Dunkirk port	6	Dunkirk	51.0484	2.2962			3		3								
Total	140																

Note

The region indicated with (*) is located in the indigenous range of *M. leidyi*.

### 2.2 Library preparation

Tissue aliquots of ca. 5 mg were dried for 15 min at 37°C to ensure ethanol evaporation. Dried tissue samples were dissolved in 50 µl water. The Bio‐Nobile QuickPick gDNA purification kit was used for DNA isolation of samples from 2014 onwards and the InViTek DNA isolation kit for samples from 2013 and older. DNA integrity was checked by gel electrophoresis, and the concentration was measured with QuantiFluor intercalating dye on a Promega Quantus fluorometer (Promega). All samples were genotyped individually with a single‐enzyme GBS protocol modified from Elshire et al. (2011). The protocol was optimized for *M. leidyi* during a pilot experiment. We compared the performance of six restriction enzymes with different recognition sites: *Mse*I (T|TAA), *Msp*I (C|CGG), *ApeK*I (G|CWGC), *EcoR*I (G|AATTC), *EcoT22*I (ATGCA|T), and *Pst*I (CTGCA|G). Ten libraries were prepared for each enzyme using a set of ten specimens. The performance of the enzymes was evaluated by gel electrophoresis of the restriction digest and a pilot sequencing run. *Msp*I was selected for genotyping all other samples (see Section 3). In short, 100 ng of genomic DNA was digested, and adapters were ligated with T4 DNA ligase. A barcode adapter—common adapter system was used with in‐line barcode sequences. Adapter sequences were generated with the tool from Deena Bioinformatics (http://www.deenabio.com/services/gbs-adapters). Barcode sequences were 4 to 9 bp, differed from each other by at least 3 substitutions and contain homopolymers of maximum 2 bp. Restriction digests and adapter ligations were performed according to the enzyme manufacturer's recommendations (New England Biolabs). Individual libraries were purified with 1.6× MagNa magnetic beads (Rohland & Reich, 2012) and eluted in 50 µl 0.1× TE buffer. Short fragments were amplified by PCR with 2 µl ligate with Taq 2× Master Mix (New England Biolabs), using a denaturation step of 5 min at 72°C + 30 s at 98°C followed by 20 cycles of 10 s at 98°C + 30 s at 65°C + 30 s at 72°C. PCR products were purified with 1.6× MagNa magnetic beads and eluted in 30 µl 0.1× TE buffer. Fragment size distributions of individual libraries were evaluated with a Qiagen QIAxcel system (Qiagen). The libraries were normalized and pooled for sequencing. The libraries of the pilot experiment were paired‐end sequenced for 100 bp on an Illumina HiSeq2000 instrument by BGI. Because of the sequencing of short fragment sizes, only the forward reads were used for the data analysis. For the second sequencing run, libraries were prepared for 175 specimens, and single‐end sequenced for 100 bp on an Illumina HiSeq 2500 instrument by the Genomic Services Lab at HudsonAlpha, using three sequencing lanes.

### 2.3 Read filtering and mapping

*GNU parallel* (Tange, 2011) was used for parallelization of all following steps. Reads were demultiplexed with *GBSX 1.1.5* (Herten, Hestand, Vermeesch, & Houdt, 2015), allowing 1 mismatch in the barcode. Common adapter sequences, restriction site remnants, and intact restriction sites were trimmed with *cutadapt 1.9.1* (Martin, 2011). Reads containing ambiguous bases and reads with an average base quality below 30 were discarded with *prinseq‐lite 0.20.4* (Schmieder & Edwards, 2011). Sequence quality was checked with *FastQC 11.7* (Andrews, 2010) and *MultiQC 1.5* (Ewels, Magnusson, Lundin, & Käller, 2016).

Quality‐filtered reads were aligned to the reference genome (Ryan et al., 2013) with the *BWA‐mem* algorithm in *BWA 0.7.17* (Li & Durbin, 2009). Mapped reads were filtered on minimum mapping quality 20, and supplementary reads were removed with *SAMtools 1.8*. (Li et al., 2009). Further analysis and filtering of the alignments were performed with custom *AWK* and *R (3.4.3)* scripts (R Core Team, 2017). To quantify the number of distinct GBS loci, we delineated stacks of reads with identical mapping positions. Stacks with read depth below six were flagged as missing data, and stacks with partially overlapping positions were grouped. Genomic coordinates of these features were stored in GFF format. We counted the number of loci per library as a function of the number of reads mapped per library (Figure S1a). Out of the 10 libraries created per enzyme, we selected the five libraries with the highest read count for that enzyme. To evaluate the completeness of datasets generated with different restriction enzymes, we analyzed for each enzyme the number of loci shared between samples using the five libraries with the highest read count (Figure S1b). We excluded samples with less than 40 k loci for genotyping of the sample collection (Figure S1c).

### 2.4 Genotype calling

Genotypes were called with *GATK 3.7* using the *HaplotypeCaller* (McKenna et al., 2010). Multi‐allelic SNPs and indels were removed with *VCFtools 0.1.14* (Danecek et al., 2011). The VCF file was annotated with the coordinates of GBS loci and predicted genes and exons (Ryan et al., 2013), using *BEDtools 2.25.0* (Quinlan & Hall, 2010). With a custom *python* script, we converted the annotated VCF file to a simpler table format for subsequent analyses in *R*. SNP positions with less than 80% of the individuals covered after the *GATK* analysis were discarded. We evaluated Hardy–Weinberg Equilibrium (HWE) with the exact test implemented in *Plink 1.9* (Chang et al., 2015) and removed SNPs with an excessive proportion of heterozygous genotype calls across all samples (*p*‐value HWE < 0.01 and *H*_o_ > *H*_e_; Figure S2). Deviating SNPs with low *H*_o_ may be fixed variants and were kept.

A substantial portion of our SNP dataset consisted of low‐frequency alleles. These are usually discarded for population analysis, because they are deemed uninformative and may contain errors (Roesti, Salzburger, & Berner, 2012). However, careful consideration of MAF filtering was recently recommended (Linck & Battey, 2019). Therefore, we compared the distribution of alleles over the geographical regions for MAF thresholds of minimum 1 and 5% (results not shown). Low‐frequency alleles in our datasets (between 1% and 5%) were unevenly distributed over the geographical regions. Therefore, these alleles represented relevant genetic diversity and we applied a MAF threshold of minimum 1% (Figure S3).

### 2.5 Outlier detection

To find SNPs that might be under natural selection, or linked to such loci, we used outlier detection. Individual outlier detection methods differ in their ability to identify outliers, and a combination of methods is recommended (Narum & Hess, 2011; Villemereuil, Frichot, Bazin, François, & Gaggiotti, 2014). We performed the PCA‐based outlier test implemented in *PCAdapt 4.0.1* (Luu, Bazin, & Blum, 2017) and the *F*_ST_ outlier test implemented in *OutFLANK 0.1* (Whitlock & Lotterhos, 2015). *PCAdapt* correlates the genotypes of SNPs with principal components (PC) and does not require population priors. Clustering of the zero‐inflated genotype data was reduced by applying the SNP thinning algorithm, with default window size and *R* ^2^ threshold. The test statistic is based on regression of the scaled genotypes and *k* PCs that represent relevant population structure. The distribution of this test statistic is expected to follow a chi‐square distribution with *k* degrees of freedom when there are no outliers present. The genomic inflation factor (GIF) is the ratio of the observed and expected median of the test statistic, and can be used for rescaling inflated distributions. The *F*_ST_ statistic of the analysis with *OutFLANK* was based on the geographical regions as prior populations (Table 1). The tails of the *F*_ST_ distribution can be trimmed before parameterization. For both methods, we corrected for multiple testing with a threshold of 0.10 for false discovery rate (FDR; Benjamini & Hochberg, 1995). The *q*‐values for the *PCAdapt* analysis were calculated using the *qvalue* package *2.8.0* (Dabney, Storey, & Warnes, 2010). The neutral panel consisted of SNPs that were flagged as nonoutlier by both methods. To examine the distribution of outliers within the nonindigenous range in more detail, we repeated the analysis for a dataset excluding specimens from Chesapeake Bay.

### 2.6 Population genetic analysis

The mean allelic richness and mean expected and observed heterozygosity (*A*_r_, *H*_e_ and *H*_o_) were calculated for the geographical regions (Table 1) using *Hierfstat 0.04‐22* (Goudet, 2005). Population structure was analyzed using the panel of all SNPs and the panel of neutral SNPs, both of datasets including and excluding Chesapeake Bay. Analysis of molecular variance (AMOVA) based on Nei's genetic distance (Nei, 1972) was performed with *pegas 0.10* (Paradis, 2010) and *StAMPP 1.5.1* (Pembleton, Cogan, & Forster, 2013), using 100 permutations. Pairwise genetic differentiation between the regions was estimated with the unbiased *F*_ST_ estimator *θ* (Weir & Cockerham, 1984), and pairwise 95% confidence intervals were calculated with *StAMPP* using 100 bootstraps. Negative *F*_ST_ values were set to zero. The population structure and population assignment of individuals were further described with discriminant analysis of principle components (DAPC) implemented in *Adegenet 2.1.0* (Jombart, 2008), using 20 PCs.

## 3 RESULTS

### 3.1 Optimization of GBS genotyping in *M. leidyi*

Of the six restriction enzymes tested, *EcoT22*I and *EcoR*I were excluded because gel electrophoresis showed insufficient DNA fragmentation. *Mse*I was excluded because the sequence reads had very low GC content (approximately 35% on average). The read depth distribution of the three remaining enzymes was evaluated either using saturation curves showing the number of loci per sample as a function of the number of reads mapped per library or, alternatively, by analyzing the number of loci shared between libraries (Figure S1a). *Pst*I yielded less than approximately 5 k GBS loci per library, independent of the number of reads used for mapping, while for *ApeK*I and *Msp*I increasingly more loci were detected above the read depth threshold (6 reads) with increasing total number of reads mapped. These curves show that about 30 k loci are expected if at least 600 k reads per library are mapped, but that mapping higher numbers of reads may be required to reach saturation in the number of independent GBS loci for *ApeK*I or *Msp*I. The number of loci shared between samples was determined per enzyme (Figure S1b). These curves indicated that *Msp*I was more efficient to sequence common loci compared with *ApeK*I. Therefore, we decided to use *Msp*I for GBS profiling of the sample collection.

### 3.2 Distribution of read data and SNPs

We obtained 140 libraries with more than 40 k GBS loci (Figure S1c) and discarded 35 libraries with less than 40 k GBS loci. The 40 k loci were detected with sufficient read depth (6 reads) in at least 80% of the 140 libraries and cover ca. 3.4 Mbp of the 156 Mbp reference genome sequence (2.2%). Approximately 162 k biallelic SNPs were called with *GATK*. We discarded approximately 5 k SNPs with excessive read depth (data not shown), approximately 1 k SNPs with excessive heterozygous genotype calls and significantly deviating from HWE (Figure S2), and around 82 k SNPs with a MAF below 1% (Figure S3). Of the remaining 74 k SNPs, 73% were located within the genes and 34% within the exons predicted by Ryan et al. (2013). No mitochondrial SNPs were detected.

### 3.3 Identification of outlier SNPs

**Figure 2:**
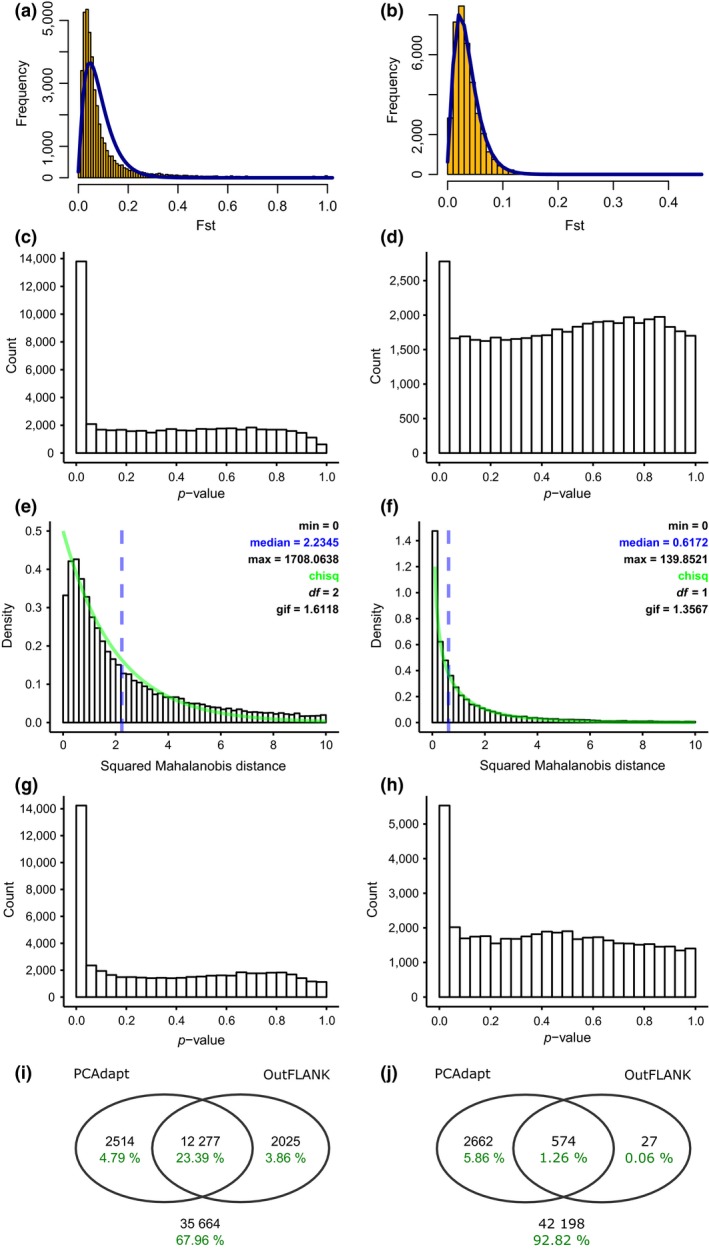
SNP outlier analysis with *OutFLANK* and *PCAdapt*. (a, b) The *F*_ST_ distributions without sample size correction generated by *OutFLANK* for, respectively, the dataset including all seven regions and the dataset without Chesapeake Bay, and (c, d) corresponding *p*‐values. (e, f) The distributions of the squared Mahalanobis distance generated by *PCAdapt* for both datasets, and (g, h) corresponding *p*‐values. (i, j) Venn diagrams show the overlap of outlier SNPs between *OutFLANK* and *PCAdapt* for both datasets, in SNP counts (black) and proportion of the SNP panels with all SNPs. The values below the Venn diagrams show the number and proportion of SNPs that were not identified as outlier by both methods (i.e., the neutral SNP panels)

The SNP thinning algorithm of *PCAdapt* reduced the SNP dataset including Chesapeake Bay from 74 to 52 k SNPs, and two relevant PCs were retained based on the clustering of samples (Figure S4a,b). The proportion of the variance captured by the first component (.17) was notably higher than the proportion captured by the second component (.02). The distribution of the test statistic was inflated (GIF 1.61); however, the fit to the expected chi‐square distribution was better without rescaling (Figure 2e). After correction for multiple testing, 14 k SNPs were identified as outliers (Figure 2i). *OutFLANK* was unable to identify outliers when using the default settings for trimming loci, because all loci with *F*_ST_ above the upper trim point were marked as outliers. To resolve this issue, we incrementally increased the fraction of highest *F*_ST_ values that was removed before parametrization of the *F*_ST_ distribution. When 30% of the highest *F*_ST_ values was removed (Figure 2a,c), 14 k outlier SNPs were identified, of which 12 k in common with *PCAdapt* (Figure 2i). Approximately 35 k SNPs (68%) were not flagged as outlier by both methods and were used as neutral SNP panel. The dataset of 69 k SNPs identified on specimens excluding Chesapeake Bay area was thinned to 45 k SNPs. *PCAdapt* recovered a single relevant PC (Figure S4c,d), and the GIF was 1.36. Again, no rescaling was applied (Figure 2f,h), and 3,236 outliers were identified (Figure 2j). *OutFLANK* was executed with default settings for trimming of loci, but very few additional outliers to *PCAdapt* were recovered (Figure 2b,d,j). For this dataset, the neutral SNP panel consisted of 42 k SNP (i.e., 93% of the SNP panel with all SNPs). For both datasets, no SNPs were flagged for having significantly low *F*_ST_ values. The proportions of SNPs located in genes and exons were similar for the panels of outlier and neutral SNPs (data not shown).

### 3.4 Genetic diversity, population structure, and population assignment of individuals

**Figure 3:**
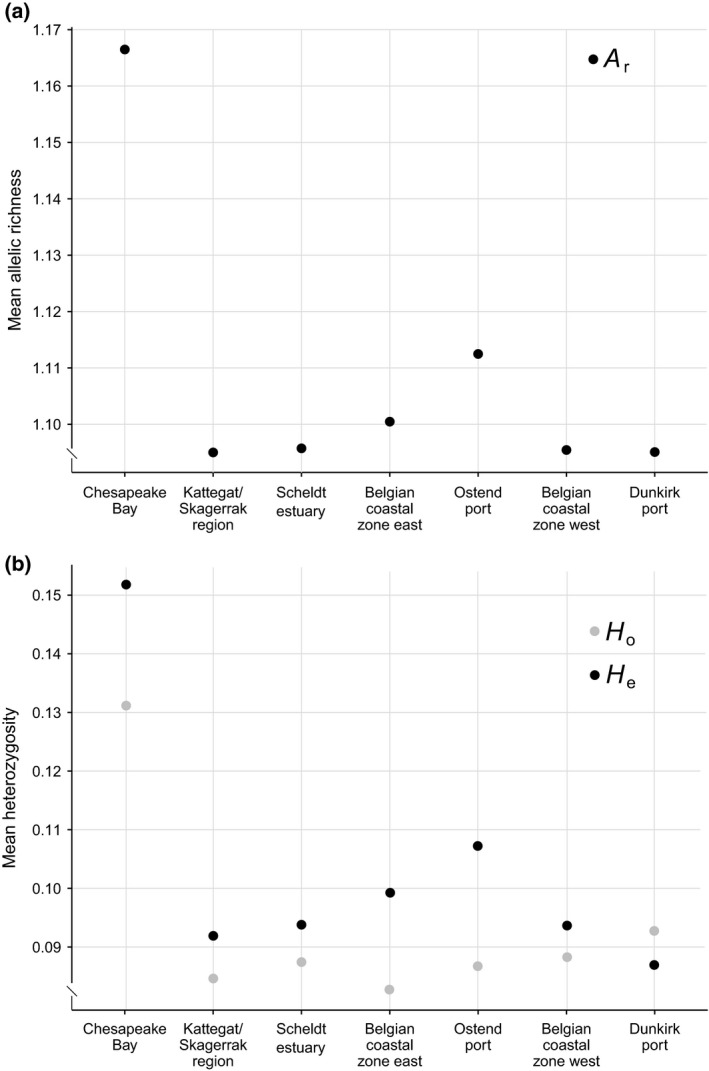
Summary statistics of genetic diversity for seven geographical regions based on 7 k SNPs with a minimum MAF of 1%, including (a) mean allelic richness (*A*_r_), and (b) mean expected (*H*_e_, black) and observed heterozygosity (*H*_o_, gray)

**Figure 4:**
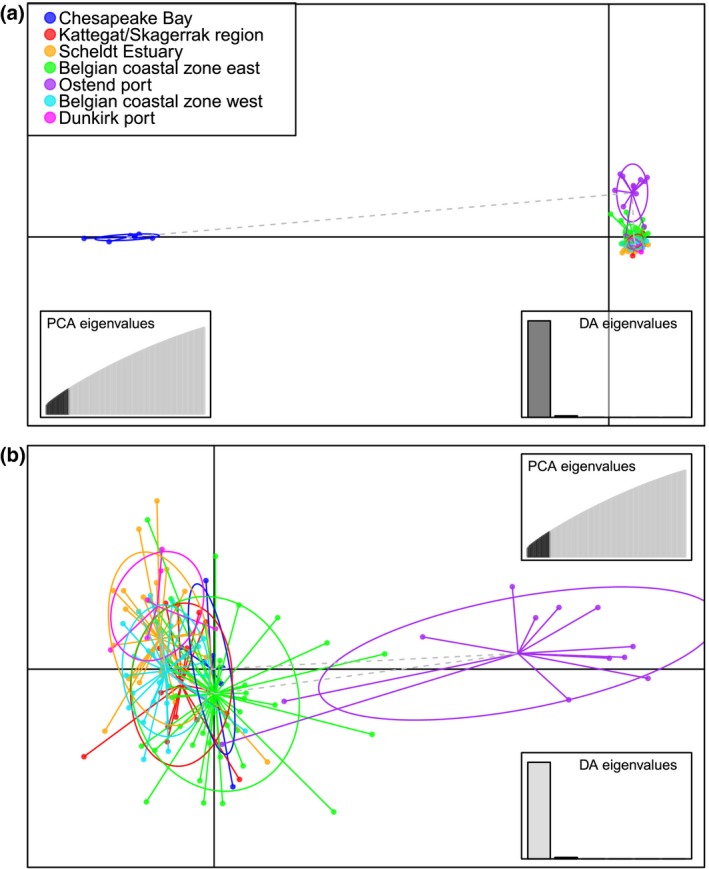
Population structure of *M. leidyi* as revealed by discriminant analysis of principal components (DAPC) based on the panel with all 74 k SNPs. (a) The first and the second discriminant, (b) the second and third discriminant. Colors indicate the geographical region of origin

**Figure 5:**
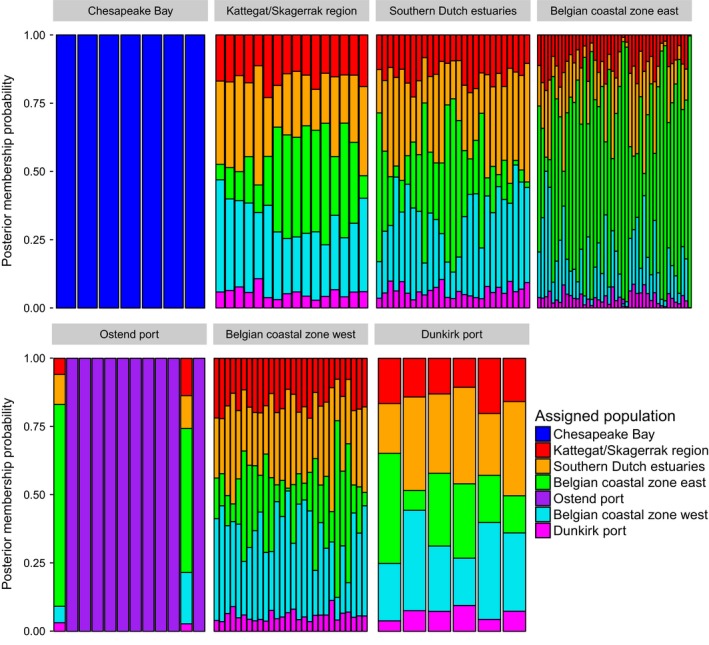
Population assignment of *M. leidyi* individuals based on DAPC of the SNP panel with all 74 k SNPs. Two individuals collected in the port of Ostend are related to the North Sea population in adjacent coastal areas

Mean *H*_e_ and *A*_r_ showed similar patterns among geographical regions (Figure 3). The highest diversity was measured for Chesapeake Bay, followed by the port of Ostend. Mean *H*_o_ was generally lower than mean *H*_e_, except for the port of Dunkirk, which also had the highest mean *H*_o_ of all nonindigenous regions. AMOVA indicated highly significant population structure between the seven regions (*p* < .001) for both panels (all SNPs or only neutral SNPs) and both the datasets including and excluding Chesapeake Bay (Table 2). Pairwise comparisons revealed significant differentiation of two regions, Chesapeake Bay and Ostend port, from all other regions (Table 3). Pairwise *F*_ST_ values between the indigenous and all nonindigenous regions ranged between .36 and .39 for all SNPs, and between .11 and .12 for neutral SNPs. Pairwise differentiation between Ostend port and all other nonindigenous regions ranged between .03 and .04 for all SNPs, and between .01 and .02 for neutral SNPs. Similar results were obtained for the dataset excluding Chesapeake Bay (result not shown). The results of DAPC based on all SNPs (Figure 4) or based on the neutral SNPs alone (Figure S5) confirm the presence of three clusters: Chesapeake Bay, Ostend port, and all other North Sea regions. The assignment of individuals to regions clearly illustrated the distinctiveness of Chesapeake Bay and Ostend (Figure 5). In contrast, the remaining five regions showed a nearly equal contribution of loci to all individuals (Figure 5). Two specimens collected in Ostend port were assigned to the North Sea cluster (Figures 4b and 5).

**Table 2:** Analysis of molecular variance (AMOVA) of the datasets including and excluding the native region Chesapeake Bay, based on the SNP panels containing all 74 k SNPs or 35 k neutral SNPs separately

	*df*	SS	MSS	Variance (%)	*σ* ^2^	*p*
Including Chesapeake Bay						
All SNPs						
Between regions	6	1.02	.170	34.3	.00835	.00
Within regions	133	1.96	.015	65.7	.01472	
Total	139	2.98	.021	100.0		
Neutral SNPs						
Between regions	6	0.29	.048	9.3	.00145	.00
Within regions	133	2.84	.021	90.7	.02139	
Total	139	3.14	.023	100.0		
Excluding Chesapeake Bay						
All SNPs						
Between regions	5	0.16	.031	6.0	.00059	.00
Within regions	127	2.45	.019	94.0	.01929	
Total	132	2.61	.020	100.0		
Neutral SNPs						
Between regions	5	0.14	.027	5.0	.00033	.00
Within regions	127	2.57	.020	95.0	.02024	
Total	132	2.71	.021	100.0		

Abbreviations: *df*, degrees of freedom; MSS, mean sum‐of‐squares; SS, sum‐of‐squares.

**Table 3:** Pairwise *F*_ST_ between regions and *p*‐values (between brackets) for all 74 k SNPs, and the neutral SNP panel of 35 k SNPs

	Chesapeake Bay	Kattegat/Skagerrak	Scheldt estuary	Belgian coastal zone east	Ostend port	Belgian coastal zone west
All SNPs						
Chesapeake Bay	–	–	–	–	–	–
Kattegat/Skagerrak	**0.38 (0.00)**	–	–	–	–	–
Scheldt estuary	**0.38 (0.00)**	0.00 (1.00)	–	–	–	–
Belgian coastal zone east	**0.38 (0.00)**	0.00 (1.00)	0.00 (0.02)	–	–	–
Ostend port	**0.36 (0.00)**	**0.04 (0.00)**	**0.04 (0.00)**	**0.03 (0.00)**	–	–
Belgian coastal zone west	**0.39 (0.00)**	0.00 (0.33)	0.00 (0.00)	0.00 (0.37)	**0.04 (0.00)**	–
Dunkirk port	**0.37 (0.00)**	0.00 (1.00)	0.00 (1.00)	0.00 (1.00)	**0.03 (0.00)**	0.00 (1.00)
Neutral SNPs						
Chesapeake Bay	–	–	–	–	–	–
Kattegat/ Skagerrak	**0.12 (0.00)**	–	–	–	–	–
Scheldt estuary	**0.12 (0.00)**	0.00 (1.00)	–	–	–	–
Belgian coastal zone east	**0.11 (0.00)**	0.00 (1.00)	0.00 (1.00)	–	–	–
Ostend port	**0.12 (0.00)**	**0.02 (0.00)**	**0.02 (0.00)**	**0.01 (0.00)**	–	–
Belgian coastal zone west	**0.12 (0.00)**	0.00 (1.00)	0.00 (0.28)	0.00 (1.00)	**0.02 (0.00)**	–
Dunkirk port	**0.12 (0.00)**	0.00 (1.00)	0.00 (1.00)	0.00 (1.00)	**0.01 (0.00)**	0.00 (1.00)

Note

Pairwise comparisons with *F*_ST_ above .01 and *p*‐value below .01 are indicated in bold.

## 4 DISCUSSION

### 4.1 Genotyping‐by‐sequencing for marine population genetics

SNPs have proven to be effective for genetic characterization of marine populations (Benestan et al., 2015; Carreras et al., 2017; Hess, Campbell, Close, Docker, & Narum, 2013), including zooplankton populations (Blanco‐Bercial & Bucklin, 2016; Reitzel, Herrera, Layden, Martindale, & Shank, 2013) and invasive species (Jeffery et al., 2017; Tepolt & Palumbi, 2015). High‐density markers outperform other analytical techniques in detecting population genetic structure (Bradbury et al., 2015; Jeffries et al., 2016). Estimating an optimal marker density in advance is difficult, especially when genetic diversity and population history are unknown. Therefore, a dense genomic distribution of SNPs is preferred (Catchen et al., 2017; Lowry et al., 2017). We optimized a one‐enzyme GBS procedure (Elshire et al., 2011) for *M. leidyi* by comparing the performance of six restriction enzymes and opted for the frequent cutting enzyme *Msp*I, based on marker density and data completeness.

### 4.2 Population connectivity among environmentally distinct regions in the Southern part of the North Sea

The recurrent presence of *M. leidyi* in the Southern part of the North Sea indicates the establishment of a persistent population (Vansteenbrugge et al., 2015). *Mnemiopsis leidyi* has been observed during the winter in the Scheldt estuary and ports along the Belgian coast, and the presence of larvae and high population densities suggested that reproduction events in these areas might populate the adjacent coastal zone (Vansteenbrugge et al., 2015). Our data provide clear evidence for the presence of two genetically distinct clusters in the Southern part of the North Sea. The first cluster represents a broadly distributed population, present in the Scheldt Estuary, the Belgian coastal zone, and Dunkirk port and is consistent with the suggested exchange of individuals between the Scheldt estuary, ports, and the North Sea. The second cluster represents a secluded population that was only sampled in the port of Ostend.

Adaptation to the local environment is an important driver of population differentiation in marine organisms (Gagnaire et al., 2015). However, since the population structure based on the neutral SNP panels was highly significant, much of the differentiation between these two clusters was driven by neutral processes. Genetic drift and limited gene flow due to physical barriers (e.g., sluices) might promote local differentiation of the Ostend port population. However, this port was not completely isolated as we identified two individuals of the North Sea population within this location. Also, fluctuating population size of the Ostend port population might reduce the effective population size and promote neutral differentiation (Kalinowski & Waples, 2002; Wright, 1938). It is unlikely that genetic bottlenecks recently occurred since the Ostend port population was more genetically diverse than any other region sampled in the indigenous range. Alternatively, recent reintroduction from distant populations or secondary spread from within the nonindigenous range might explain the difference between both genetic clusters in the Southern part of the North Sea.

Our data further show that Ostend port is probably not an important source area for the North sea, because no individuals collected in the adjacent coastal area were assigned to this population. The two other potential source areas, the Scheldt Estuary and Dunkirk port, were inhabited by the prevalent genetic cluster and are better candidate source populations. However, we did not detect a higher genetic diversity in the Scheldt Estuary and Dunkirk port compared to the coastal area. This can be explained by population expansion during the seasonal reproduction events.

### 4.3 Population connectivity between geographically distant regions of the North Sea and Baltic Sea

We investigated the population structure of *M. leidyi* over a large north–south range across the North Sea. We compared specimens collected in the Southern part of the North Sea in 2014 with specimens of the Kattegat/Skagerrak region that were collected in the same year. Although these regions are 500 km apart, both groups were indistinguishable. The lack of population differentiation in marine invertebrates is commonly explained by large population sizes (Deagle et al., 2015) and population connectivity due to limited physical barriers (Cowen & Sponaugle, 2009). Alternatively, similar invasion histories can also explain limited population differentiation between the nonindigenous regions. In the indigenous species range of *M. leidyi*, patterns of population subdivision concord with well‐established oceanographic boundaries. Distinct populations were found along the coastal zones north and south of the oceanographic front of Cape Hatteras (ca. 550 km apart; Bayha et al., 2015). Also, long‐term survival and dispersal in the open ocean were described in the indigenous species range (Bayha et al., 2015). Secondary spread via ocean currents is possibly an important process maintaining homogeneity (David et al., 2015; Grosholz, 1996). Recent expansion of the prevalent population type toward the Baltic Sea is supported by the local extinction of a genetically distinct population in the Baltic Sea population during 2012 and 2013 (Bolte et al., 2013; Jaspers et al., 2018). Last, anthropogenic transport might play an important role for invasive species (Pérez‐Portela, Arranz, Rius, & Turon, 2013). Transport via ballast water between the North and Baltic Sea is likely to occur, as the area is part of a heavily trafficked maritime transport network (Kaluza, Kölzsch, Gastner, & Blasius, 2010). Rapid recolonization of nonindigenous areas after local population extinction, either by natural dispersal via ocean currents or human‐mediated reintroduction, contributes to the invasive success of *M. leidyi*.

### 4.4 The introduction of *M. leidyi* in Northwestern Europe

Previous molecular studies of *M. leidyi* showed that the Northwestern European invasion originated from the indigenous species range along the Atlantic coast of North America. Nonindigenous populations often experience decreased genetic diversity due to founder effects. The ability to self‐fertilize and establish new populations in nonindigenous areas with an extremely small number of individuals could intensify this phenomenon. However, previous studies found no evidence of decreased genetic diversity of *M. leidyi* in the Northwestern Europe. This was attributed to the introduction of large numbers of ctenophores, possibly from recurrent introductions (Bayha et al., 2015; Ghabooli et al., 2011; Reusch et al., 2010). This issue was reevaluated in this study using high‐density molecular markers.

We showed the presence of extensive population structure across the North Atlantic Ocean, with significant neutral differentiation between all individuals of the nonindigenous area of the North Sea and the indigenous population of Chesapeake Bay. Furthermore, genetic diversity among regions in the nonindigenous range was lower than in the indigenous population, despite the much lower sampling size in the latter population. This suggests founder effects and limited reintroduction pressure from the indigenous range. However, these results should be interpreted with care. First, comparison among studies is complicated, since sampling dates span several years. The population in Chesapeake Bay may no longer represent the original population introduced in Northwestern Europe. This scenario is likely because local extinction and recolonization were also observed in the nonindigenous range (Jaspers et al., 2018). Secondly, it should be noted that our sample collection included only seven specimens from a single location, while reconstruction of the invasion histories and the identification of native source populations require extensive sampling in the indigenous species range (Gaither, Bowen, & Toonen, 2013; Muirhead et al., 2008).

## 5 CONCLUSION

Our results show that GBS is a powerful method to investigate fine‐scale structure among *M. leidyi* populations. We found evidence for the presence of two genetically distinct populations in the North Sea, with a secluded population present in a single port and a prevalent population present in environmentally distinct and geographically distant regions of the North Sea. Dispersal via ocean currents and anthropogenic transport are important factors in secondary spread of *M. leidyi* and a better understanding of these mechanisms is crucial for developing effective control measures and management strategies. Both nonindigenous populations were genetically distinct from a population sampled in the indigenous species range. More elaborate sampling in both the indigenous and the nonindigenous area would deepen our insight of introduction pathways and establishment of *M. leidyi*.

## CONFLICT OF INTEREST

None declared.

## AUTHOR CONTRIBUTION

CV, LV, HM, OH, TR, IRR, and KH planned the experiment. CV and TK carried out the laboratory experiments. CV and TR analyzed the NGS data. CV, LV, SD, and KH conducted the population genetic analysis. CV, LV, SD, OH, TR, IRR, and KH wrote the manuscript. All authors read and approved the final manuscript.

## Data Availability

The DNA sequences generated for this study are available at the NCBI sequence read archive under SRA accession numbers SRR6950721–SRR6950884.
