# Supplementary material for: Population genomic structure of the gelatinous zooplankton species Mnemiopsis leidyi in its nonindigenous range in the North Sea
Source: Ecol Evol. 2019 Sep 30;10(1):11–25. doi: 10.1002/ece3.5468 (PMC6972810; doi:10.1002/ece3.5468)
Supplement: Supplementary file 1 [file ECE3-10-11-s001.docx]

# Supporting Information


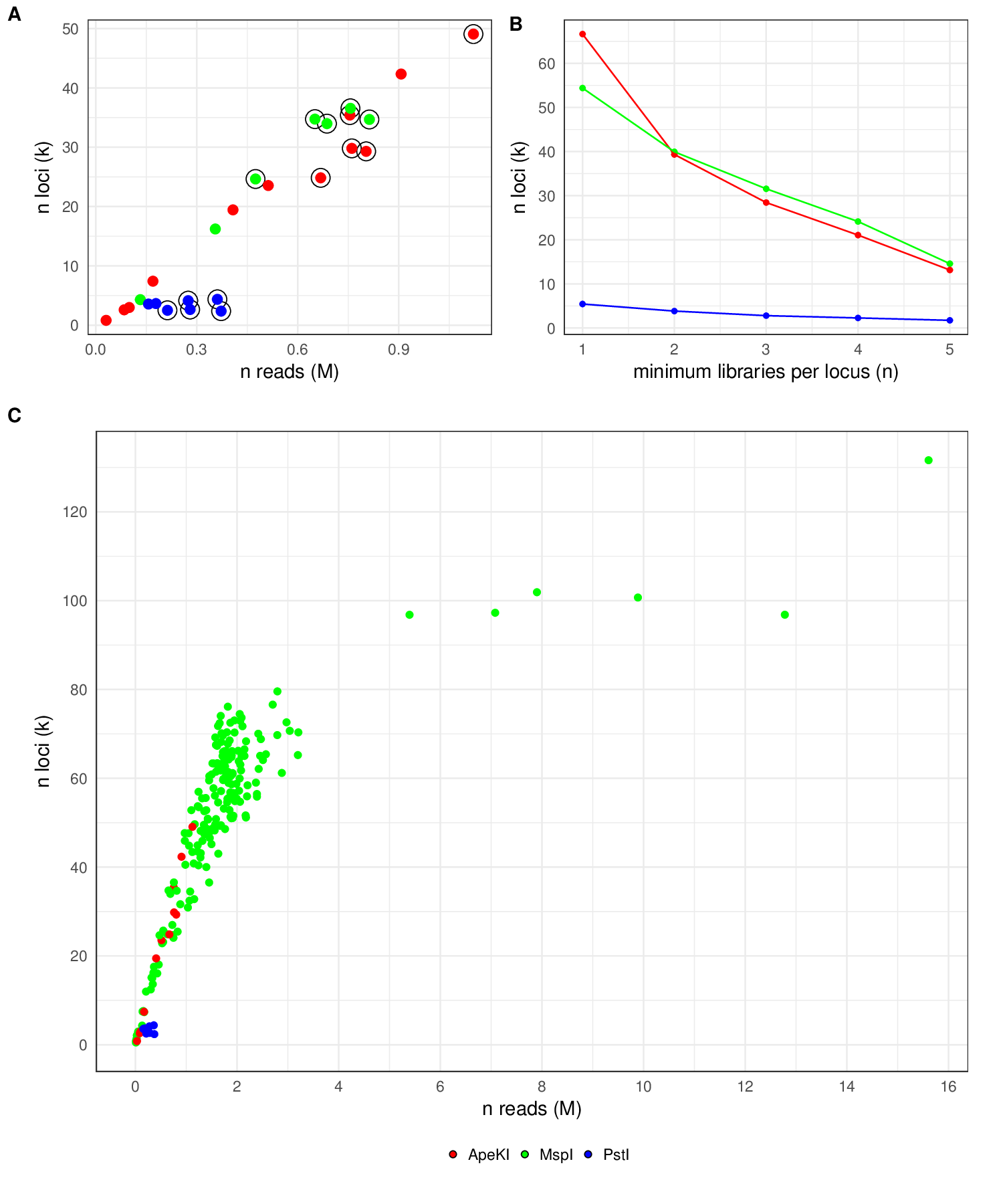


Figure S1 Analysis of read depth distribution (A) The saturation curve of the pilot experiment shows the number of loci per library (with minimum 6 reads) in function of number of mapped reads per library. Colors represent the restriction enzymes; *Pst*I (blue), *ApeK*I (red), and *Msp*I (green). *PstI* yielded maximum 5 k GBS loci, and was outperformed by the other enzymes that yielded 30 k loci if 600 k reads are mapped. Circles indicate libraries of unique individuals with the five most read counts that were used for (B). Comparison of data completeness among restriction enzymes estimated as the number of libraries per locus. *Msp*I performs slightly better than *ApeK*I, as relatively more loci were common between libraries. (C) Saturation curve of all libraries prepared for sequencing. The majority of potentially available GBS loci were recovered if at least 600 k reads were mapped.


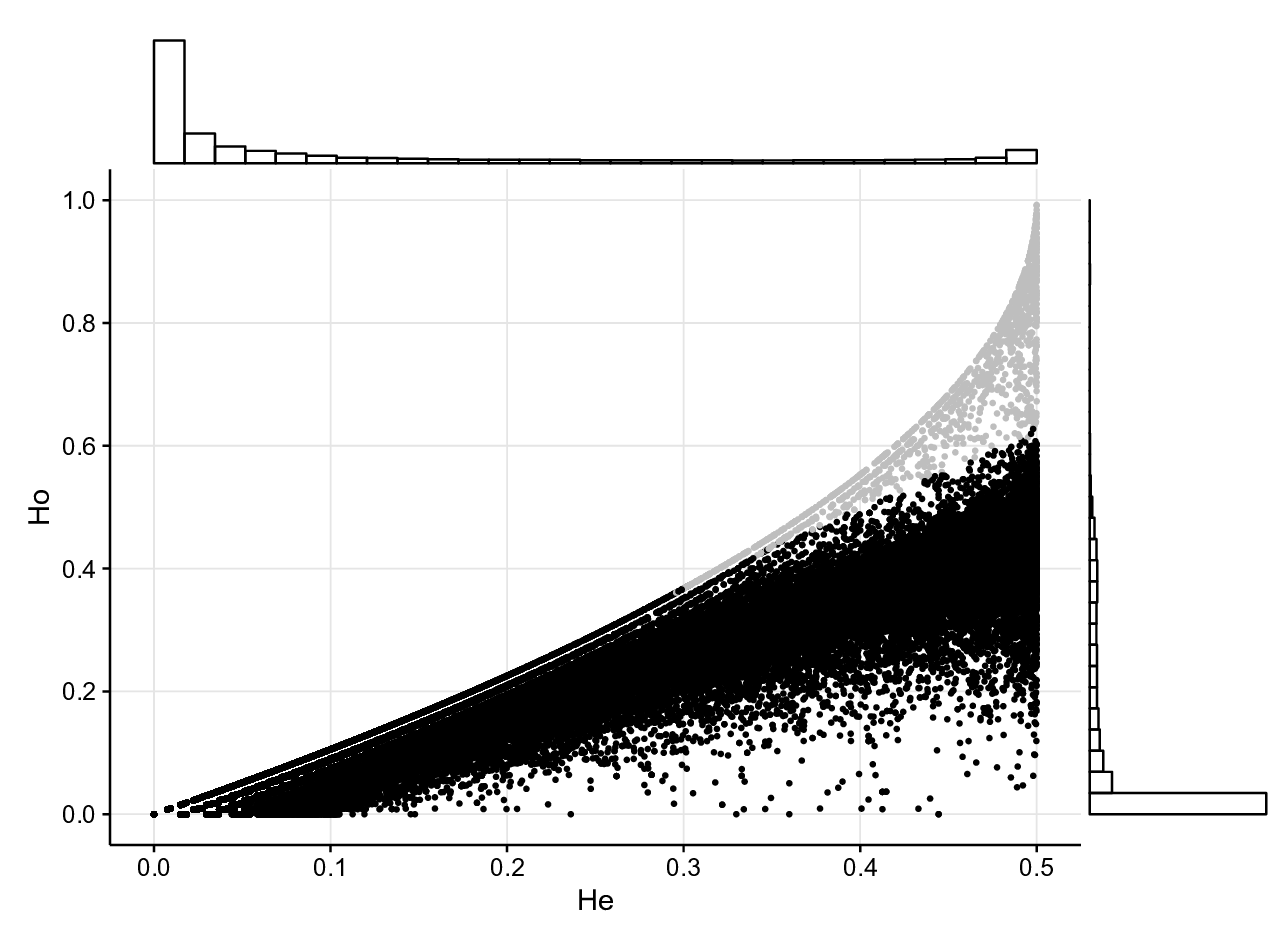


Figure S2 Relation between the expected ($H_{e}$) and observed heterozygosity ($H_{o}$) for 175 k SNPs. We discarded approximately 1 k SNPs that significantly deviated from Hardy-Weinberg Equilibrium (HWE) (p < 0.01; $H_{e}$ < $H_{o}$; grey dots).


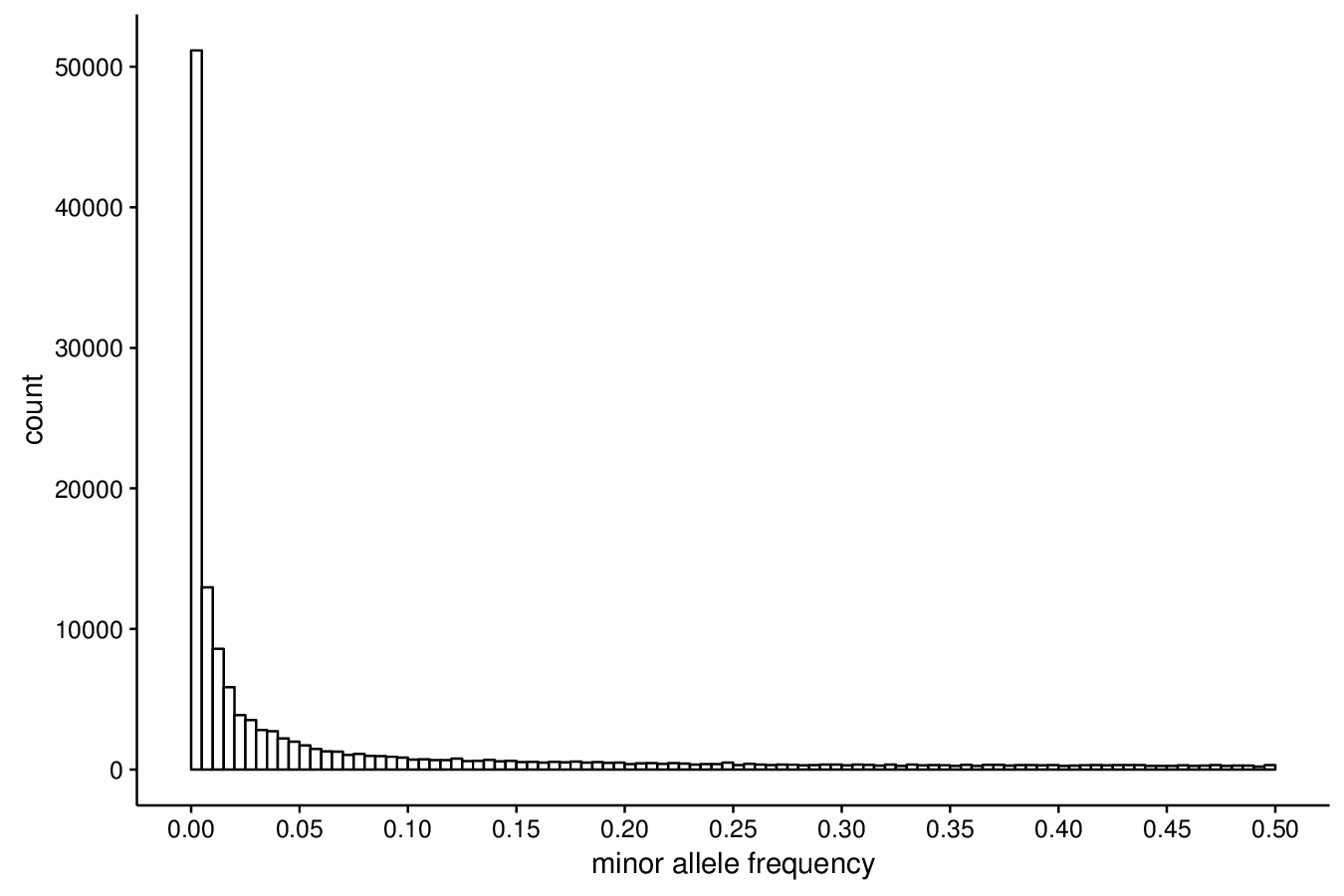


Figure S3 Alternative allele frequency distribution of 74 k SNPs among 140 *M. leidyi* individuals.


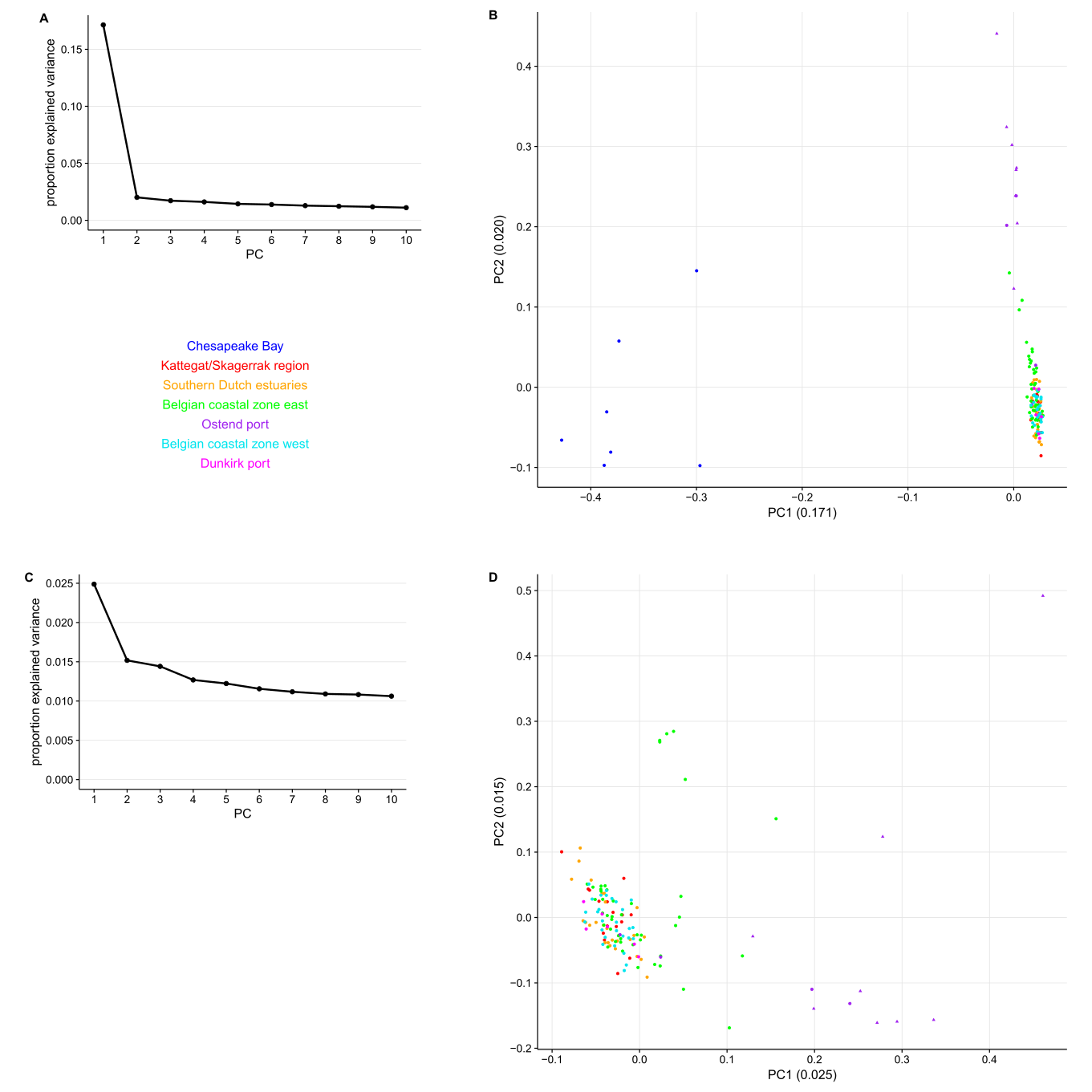


Figure S4 Population structure of *M. leidyi* as revealed by principal component analysis (PCA) (implemented in *PCAdapt*) based on SNPs with min. MAF of 3%, and centered and scaled genotypes (A) and (B) respectively show the screeplot and scores of the first two PCs for the dataset including all seven regions (140 individuals). (C) and (D) respectively show the scree plot and scores of the first two PCs for the dataset including non-indigenous regions only (133 individuals). Colors represent the region of origin, triangles represent individuals collected in 2015, and dots represent individuals collected before 2015.


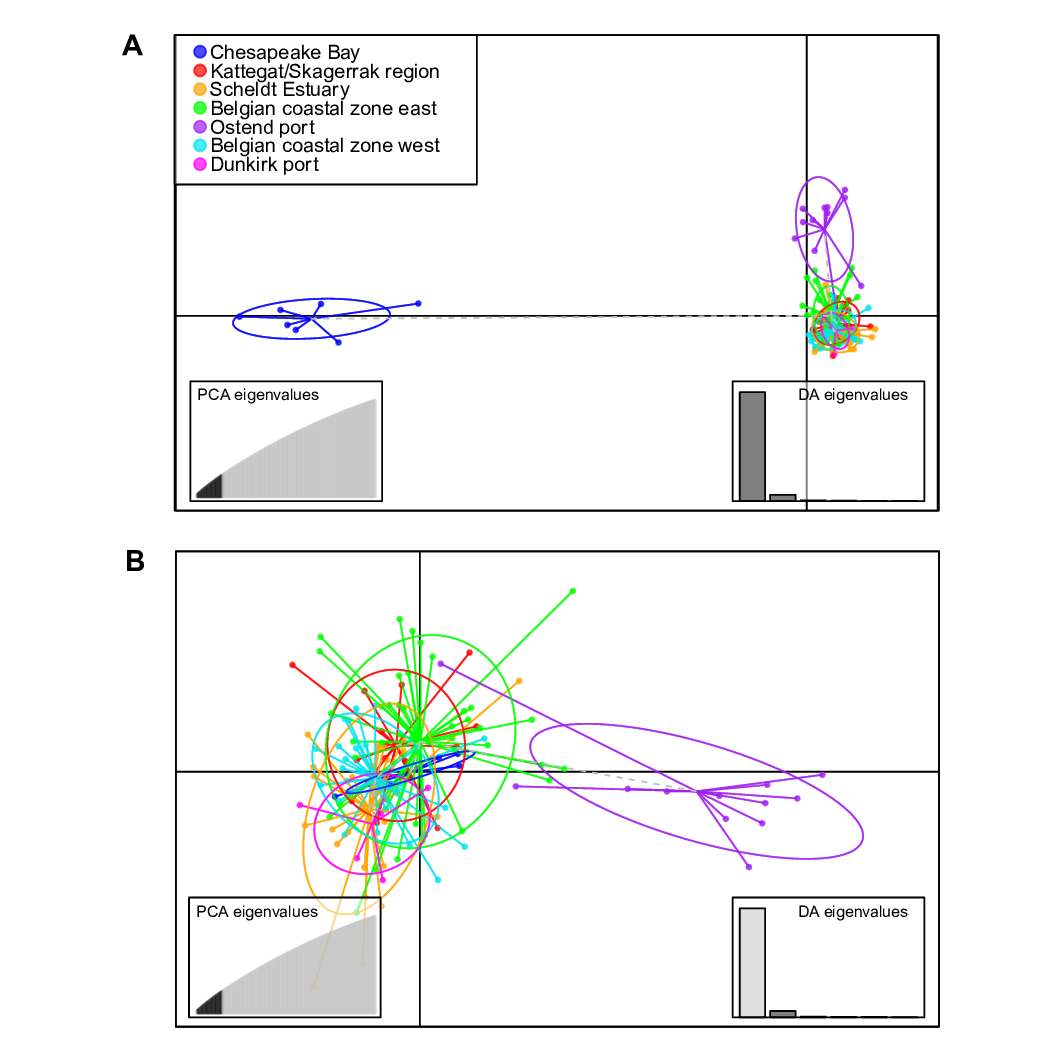


Figure S5 Population structure described with DAPC of the SNP panel with 35 k neutral SNPs for the dataset including Chesapeake Bay. (A) First and second discriminant, (B) second and third discriminant. Colors indicate the geographical region of origin.
